# Tumour necrosis as assessed with ^18^F-FDG PET is a potential prognostic marker in diffuse large B cell lymphoma independent of *MYC* rearrangements

**DOI:** 10.1007/s00330-019-06178-9

**Published:** 2019-04-26

**Authors:** Xaver U. Kahle, Menno Hovingh, Walter Noordzij, Annika Seitz, Arjan Diepstra, Lydia Visser, Anke van den Berg, Tom van Meerten, Gerwin Huls, Ronald Boellaard, Thomas C. Kwee, Marcel Nijland

**Affiliations:** 1 Department of Hematology, University of Groningen, University Medical Center Groningen, Groningen, The Netherlands; 2 Department of Nuclear Medicine and Molecular Imaging, University of Groningen, University Medical Center Groningen, Groningen, The Netherlands; 3 Department of Pathology and Medical Biology, University of Groningen, University Medical Center Groningen, Groningen, The Netherlands; 4 Department of Radiology, University of Groningen, University Medical Center Groningen, Groningen, The Netherlands

**Keywords:** Diffuse, large B cell, lymphoma, MYC oncogene, Necrosis, Fluorodeoxyglucose F18, Positron emission tomography

## Abstract

**Objectives:**

*MYC* gene rearrangements in diffuse large B cell lymphomas (DLBCLs) result in high proliferation rates and are associated with a poor prognosis. Strong proliferation is associated with high metabolic demand and tumour necrosis. The aim of this study was to investigate differences in the presence of necrosis and semiquantitative ^18^F-FDG PET metrics between DLBCL cases with or without a *MYC* rearrangement. The prognostic impact of necrosis and semiquantitative ^18^F-FDG PET parameters was investigated in an explorative survival analysis.

**Methods:**

Fluorescence in situ hybridisation analysis for *MYC* rearrangements, visual assesment, semiquantitative analysis of ^18^F-FDG PET scans and patient survival analysis were performed in 61 DLBCL patients, treated at a single referral hospital between 2008 and 2015.

**Results:**

Of 61 tumours, 21 (34%) had a *MYC* rearrangement (*MYC*^*+*^). *MYC* status was neither associated with the presence of necrosis on ^18^F-FDG PET scans (necrosis^PET^; *p* = 1.0) nor associated with the investigated semiquantitative parameters maximum standard uptake value (SUV_max_; *p* = 0.43), single highest SUV_max_ (*p* = 0.49), metabolic active tumour volume (MATV; *p* = 0.68) or total lesion glycolysis (TLG; *p* = 0.62). A multivariate patient survival analysis of the entire cohort showed necrosis^PET^ as an independent prognostic marker for disease-specific survival (DSS) (HR = 13.9; 95% CI 3.0–65; *p* = 0.001).

**Conclusions:**

*MYC* rearrangements in DLBCL have no influence on the visual parameter necrosis^PET^ or the semi-quantiative parameters SUV_max_, MATV and TLG. Irrespective of *MYC* rearrangements, necrosis^PET^ is an independent, adverse prognostic factor for DSS.

**Key Points:**

*• Retrospective analysis indicates that MYC rearrangement is not associated with necrosis on* ^*18*^ *F-FDG PET (necrosis* ^*PET*^ *) scans or semiquantitative* ^*18*^ *F-FDG PET parameters.*

*• Necrosis* ^*PET*^ *is a potential independent adverse prognostic factor for disease-specific survival in patients with DLBCL and is not influenced by the presence of MYC rearrangements.*

**Electronic supplementary material:**

The online version of this article (10.1007/s00330-019-06178-9) contains supplementary material, which is available to authorized users.

## Introduction

Diffuse large B cell lymphoma (DLBCL) accounts for 35% of all B cell non-Hodgkin lymphomas (B-NHL) [1]. Approximately 10–15% of DLBCL cases harbour a *MYC* gene rearrangement (*MYC*^*+*^), as assessed by fluorescence in situ hybridisation (FISH) [2]. These lymphomas are characterised by a very high proliferation rate. Patients bearing a *MYC*^*+*^ lymphoma experience an aggressive clinical course and have a poor prognosis when treated with the standard regimen of rituximab, cyclophosphamide, doxorubicin, vincristine and prednisolone (R-CHOP) [3]. In 2017, the World Health Organization (WHO) established a new entity for *MYC* rearranged DLBCL, called ‘high-grade B-cell lymphoma with *MYC* and *BCL2* and/or *BCL6* rearrangements’ [1, 4].

MYC is an oncogenic transcription factor regulating a vast array of cellular processes and pathways [5, 6]. Tumour cells overexpressing MYC meet their high energy demands by increased glucose uptake, glycolysis, lactate production and amino acid consumption [7, 8]. However, unlike physiological tissues, cancer cells frequently have acquired resistance to apoptosis and cannot regulate their energy expenditure during metabolic stress, resulting in cell death via necrosis when nutrient supply is compromised [9–11].

In B-NHL patients, ^18^F-fluorodeoxyglucose positron emission tomography (^18^F-FDG PET) scans are used for staging and response assessment [12]. Tumour necrosis can be assessed by visual inspection of ^18^F-FDG PET scans (necrosis^PET^) [13]. Necrosis can be observed in 14–20% of DLBCL cases and has been associated with an adverse prognosis [14, 15]. Semiquantitative assessment of ^18^F-FDG PET allows for relative comparison of parameters based on the spatial distribution and degree of ^18^F-FDG uptake, and is currently being investigated as a tool for therapy monitoring and assessing prognosis in B-NHL [16–18]. Still, data on the prognostic value of the semiquantitative parameters maximum standardised uptake value (SUV_max_) and metabolically active tumour volume (MATV) in DLBCL are conflicting [19–21].

*MYC* rearrangement, tumour necrosis (necrosis^PET^) and parameters derived from semiquantitative analysis of ^18^F-FDG PET are fundamentally linked to metabolism, yet the relationship between these factors remains unknown. We hypothesise that the higher metabolic activity mediated by *MYC* rearrangements might result in a higher incidence of necrosis^PET^ and increased semiquantitative parameters. The previously suggested prognostic impact of necrosis^PET^ [15] and semiquantitative parameters [16–18] in DLBCL might be accredited to their potential association with *MYC* rearrangements.

Therefore, the aim of this study was to investigate differences in the presence of necrosis^PET^ and semiquantitative ^18^F-FDG PET metrics between DLBCL cases with or without a *MYC* rearrangement. The prognostic impact of these factors was explored by means of survival analysis.

## Materials and methods

### Study design and case selection

**Fig. 1:**
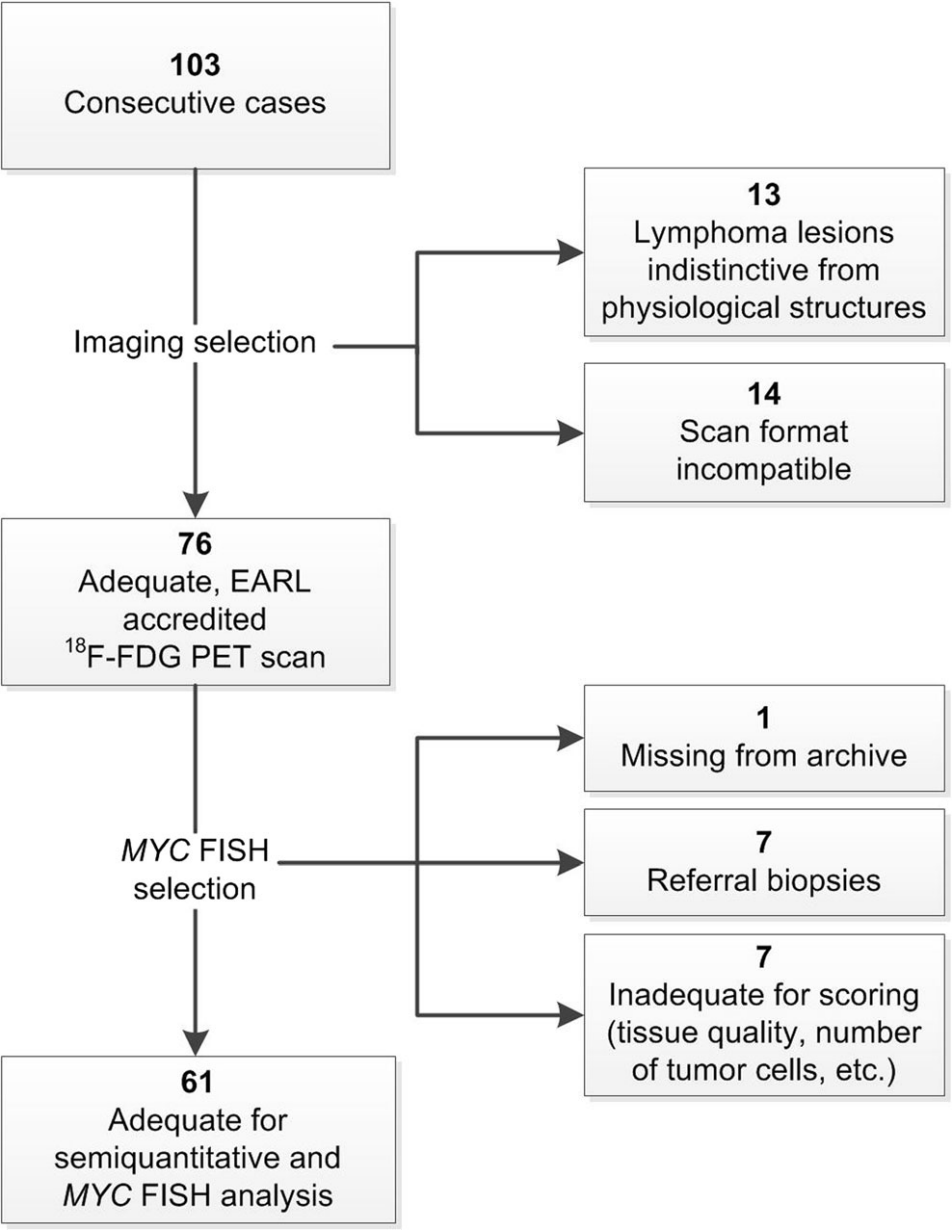
Flow-chart of case selection

For this retrospective single-centre study, consecutive patients with newly diagnosed, histologically confirmed DLBCL between 2008 and 2015 were identified in the electronic healthcare database of the University Medical Center Groningen (UMCG), a reference centre for aggressive B cell lymphomas. Cases of primary cutaneous DLBCL, primary central nervous system lymphoma, primary mediastinal B cell lymphoma and immunodeficiency-associated lymphomas were excluded. The selection of cases for this study is summarised in Fig. 1. Patients were stratified according to the National Comprehensive Cancer Network international prognostic index (NCCN-IPI) [22]. End of treatment response was assessed by ^18^F-FDG PET/CT scan. Tumour responses were classified according to Lugano criteria [12]. Follow-up was registered until early October 2017. According to Dutch regulations, no medical ethical committee approval was required for this retrospective, non-interventional study. A waiver was obtained from the medical ethics committee of the UMCG on November 13, 2018. The study utilised rest material from patients, the use of which is regulated under the code for good clinical practice in the Netherlands and does not require informed consent in accordance with Dutch regulations.

### Pathology review

Pathology review was done using the 2008 WHO classification of haematopoietic and lymphoid tissues (AD) [23]. Histological scoring for necrosis (necrosis^Hist^) was done by microscopic assessment of haematoxylin and eosin–stained slides. Only microscopic areas with definite histopathological signs of necrosis (i.e. karyolysis) were scored as positive for necrosis^Hist^.

### *MYC* fluorescence in situ hybridisation

For evaluation of a *MYC* rearrangement, formalin-fixed paraffin-embedded tissue blocks of primary tumour samples were used. Interphase fluorescence in situ hybridisation (FISH) was performed on 4-μm-thick whole tissue sections, using Vysis break apart probes (Abbot Technologies) and standard FISH protocols as previously described [24]. Researchers performing *MYC* FISH analyses were blinded for results from visual scoring, microscopic assessment of necrosis (necrosis^Hist^) and clinical outcome.

### ^18^F-FDG PET imaging

All ^18^F-FDG PET scans were performed prior to therapy. Patients were allowed to continue all medication and fasted for at least 6 h before whole-body (from the skull vertex to mid-thigh level) three-dimensional PET images were acquired. This was done 60 min after intravenous administration of a standard dose of 3 MBq/kg (0.081 mCi/kg) bodyweight ^18^F-FDG on a Biograph mCT (Siemens Healthineers), according to the European Association of Nuclear Medicine (EANM) procedure guidelines for tumour imaging with FDG PET/CT (version 2.0) [25]. Acquisition was performed in seven bed positions of 2-min emission scans for patients 60–90 kg. Patients with body weight less than 60 kg and more than 90 kg body weight were scanned with 1 min and 3 min per bed position, respectively. Low-dose transmission CT was used for attenuation correction. Low-dose CT and ^18^F-FDG PET scans were automatically fused by the use of three-dimensional fusion software (Siemens Healthineers) with manual fine adjustments. Raw data were reconstructed through ultra-high definition (Siemens Healthineers).

### Computed tomography

Diagnostic CTs were acquired via integrated ^18^F-FDG PET/CT scans according to the European Association of Nuclear Medicine (EANM) procedure guidelines for tumour imaging with FDG PET/CT (version 2.0) [25]. Bulky disease was defined as any nodal lymphoma lesion > 10 cm in coronal, axial or sagittal planes.

### ^18^F-FDG PET analysis

**Fig. 2:**
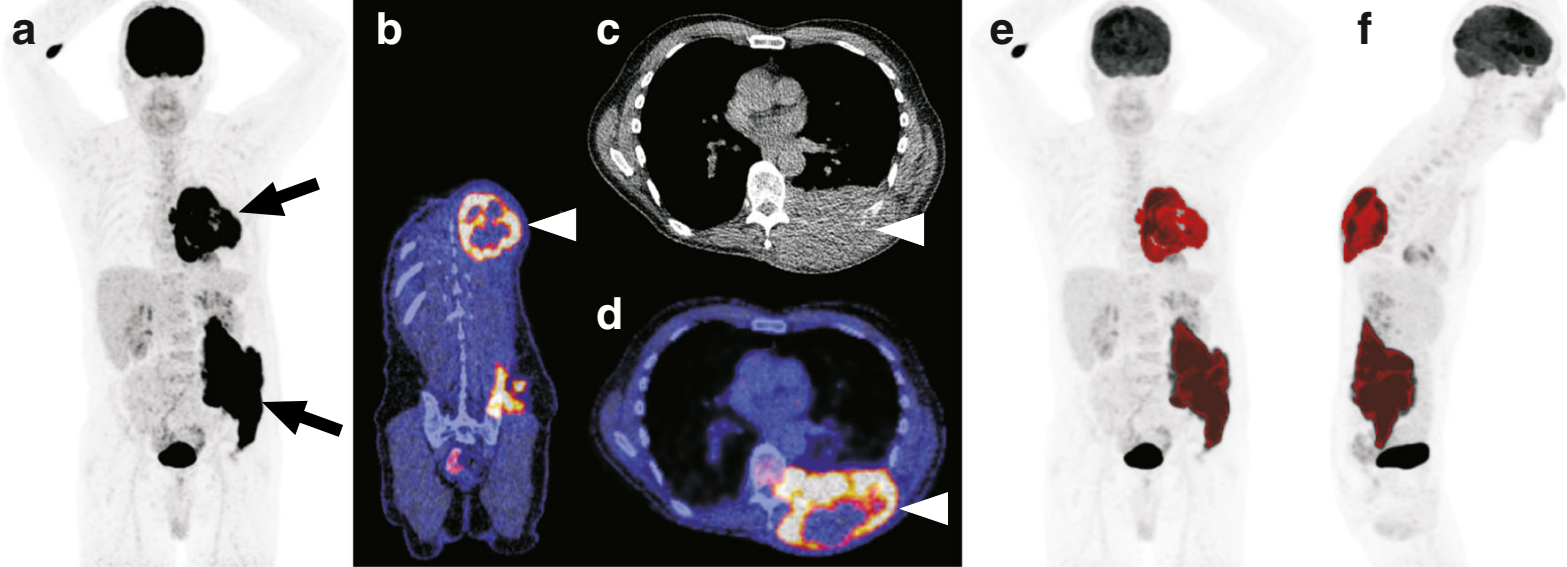
Visual assessment of necrosis and semiquantitative ^18^F-FDG PET review process. **a** A 65-year-old man with diffuse large B cell lymphoma (DLBCL) and tumour masses in the left dorsal chest wall and left pelvis, as shown on the coronal maximum intensity projection (MIP) ^18^F-FDG PET image (arrows). Coronal fused ^18^F-FDG PET/CT (**b**), axial CT (**c**) and axial fused ^18^F-FDG PET/CT (**d**) show the tumour mass with photopenic areas (arrow heads), in keeping with tumour necrosis. Coronal and sagittal MIP ^18^F-FDG PET images (**e** and **f**) show tumour segmentation (marked in red colour) for the calculation of metabolically active tumour volume (MATV), total lesion glycolysis (TLG), maximum standard uptake value (SUV_max_), and single highest SUV_max_

All ^18^F-FDG PET scans were visually assessed for the presence of tumour necrosis (necrosis^PET^) by an experienced reader (TCK), who was blinded to clinical, laboratory, biopsy and follow-up findings, as previously described [15]. Areas within any nodal or extranodal ^18^F-FDG PET–avid lymphomatous lesions that showed no ^18^F-FDG uptake were registered as having necrosis^PET^ (Fig. 2); no specific visual scale was used. Semiquantitative analysis was performed using an in-house tool for quantitative ^18^F-FDG PET/CT analysis, as previously described [26–28]. This programme automatically preselects lesions using a SUV_max_ threshold of 4 and a metabolic volume threshold of 2.5 ml. Unwanted preselected FDG-avid regions, such as the bladder and brain, are removed by user interaction. Finally, remaining FDG-avid segmentations are processed using a background-corrected 50% of SUV peak region growing method, as described by Frings et al [26], to obtain the final tumour segmentations. In case obvious lymphoma lesions were not selected (*n* = 3), they were manually added after automatic tumour segmentation. From the final segmentation, the metabolic active tumour volume (MATV, in ml), total lesion glycolysis (TLG = MATV × SUV_mean_) and SUVs are derived for each lesion independently as well as summed over all lesions. Lesion selection and semiquantitative analysis was performed by MH under direct supervision of an experienced nuclear medicine physician (WN) and a nuclear physicist (RB). SUV_max_ was defined as the highest SUV per voxel within one lymphomatous lesion. In this paper, SUV_max_ is reported as the mean of SUV_max_ across all lesions of an individual patient. SUV_max_ single highest was defined as the highest SUV_max_ of all lesions within an individual patient.

### Statistical analysis

Comparison between continuous, non-normally distributed variables was estimated by Wilcoxon rank-sum test. Differences between two nominal variables were evaluated using Pearson’s chi-square or Fisher’s exact test (for expected groups sizes ≤ 5). For exploratory survival analysis, the primary endpoints were overall survival (OS), progression-free survival (PFS) and disease-specific survival (DSS). OS was defined as the time from diagnosis until death (from any cause). PFS was defined as the time from diagnosis until death or relapse or progression [12]. DSS was defined as the time from diagnosis until death from DLBCL. Surviving patients were censored at the last date of follow-up. Survival curves were estimated according to the Kaplan-Meier method. Cox regression was used for univariate and multivariate survival analyses and results were reported as hazard ratio (HR), 95% confidence interval (CI) and *p* value based on statistical Wald test. A two-tailed *p* value of less than 0.05 indicated statistical significance. All analyses were performed using R version 3.4.1 and R-studio version 1.0.153 software.

## Results

### Patient characteristics

Characteristics of the entire cohort (61 patients) are summarised in Table 1. A total of 21 patients (34%) had a DLBCL harbouring a *MYC* rearrangement. *MYC* rearrangement was observed in 11 patients (21.6%) primarily seen in the UMCG (*n* = 51) and 10 patients (100%) referred from affiliated hospitals (*n* = 10). *MYC* groups did not differ with regard to baseline characteristics (Table 1) except for serum LDH levels, which were higher in the *MYC*-positive group (*p =* 0.036) than in cases without *MYC* rearrangement.

**Table 1:** Demographics and baseline disease characteristics of patients with diffuse large B cell lymphoma according to *MYC* status

			*MYC* status				
	Total (*n* = 61)		*MYC*^*−*^ (*n* = 40)		*MYC*^*+*^ (*n* = 21)		*p* value
	No.	%	No.	%	No.	%	
Gender							
Male	36	59.0	24	60.0	12	57.1	1.0^a^
Female	25	41.0	16	40.0	9	42.9	
Age							
Median (range)	63 (26–91)		64 (26–91)		61 (30–79)		0.64^b^
Age ≤ 60 years	24	39.3	14	35.0	10	47.6	0.5^a^
Age > 60 years	37	60.7	26	65.0	11	52.4	
Stage							
I–II	22	36.0	15	37.5	7	33.3	0.97^a^
III–IV	39	63.9	25	62.5	14	66.7	
NCCN-IPI score							
0–3	30	49.2	22	55.0	8	38.1	0.32^a^
4–8	31	50.8	18	45.0	13	61.9	
Serum LDH							
Median (range)	282 (126–3037)		237 (126–1292)		381 (140–3037)		0.04^b^
Normal	29	47.5	22	55.0	7	33.3	0.18^a^
Elevated	32	52.5	18	45.0	14	66.7	
Treatment							
R-CHOP	56	91.8	37	92.5	19	90.5	0.36^c^
Intensive chemotherapy	3	4.9	1	2.5	2	9.5	
Palliative	2	3.3	2	5.0	0	0	

^a^Pearson’s chi-square test with Yates’ continuity correction

^b^Wilcoxon rank-sum test with continuity correction

^c^Fisher’s exact test for count data

### *MYC* status, necrosis and semiquantitative ^18^F-FDG PET parameters

necrosis^PET^ was observed in 15 patients (25%). The relationships between *MYC* status and necrosis^PET^, necrosis^Hist^ and semiquantitative ^18^F-FDG PET parameters are summarised in Table 2. *MYC*^*+*^ cases did not differ from cases without *MYC* rearrangement with regard to necrosis^PET^ (*p* = 1.0) or necrosis^Hist^ (*p* = 0.52).

**Table 2:** Necrosis and semiquantitative ^18^F-FDG PET parameters according to *MYC* status

			*MYC* status				
	Total (*n* = 61)		*MYC*^*−*^ (*n* = 40)		*MYC*^*+*^ (*n* = 21)		*p* value
	No.	%	No.	%	No.	%	
necrosis^PET^							
Absent	46	75.4	30	75.0	16	76.2	1.0^c^
Present	15	24.6	10	25.0	5	23.8	
necrosis^Hist^							
Absent	42	68.9	28	70.0	14	66.7	0.52^c^
Present	16	26.2	11	27.5	5	23.8	
Not available	3	4.9	1	2.5	2	9.5	
SUV_max_							
Median (range)	13.0 (3.0–38.4)		13.1 (3.0–33.9)		10.4 (5.8–38.4)		0.43^b^
SUV_max_ single highest							
Median (range)	18.8 (3.8–45.8)		19.7 (3.8–39.0)		14.2 (5.8–45.8)		0.49^b^
MATV							
Median (range)	154.7 (1–3774)		156.0 (1–2800)		154.7 (7–3774)		0.68^b^
TLG							
Median (range)	1387.4 (3–29,462)		1632.8 (3–29,462)		1147.1 (47–20,065)		0.62^b^

^b^Wilcoxon rank-sum test

^c^Fisher’s exact test for count data

When the semiquantitative parameters SUV_max_, SUV_max_ single highest, MATV and TLG were studied, no difference between *MYC* groups was observed. There was no relation between the presence of necrosis^PET^ and necrosis^Hist^ (*p* = 0.1; Supplementary Figure 1).

### Necrosis^PET^ and tumour volume

In 14 of 15 necrosis^PET^ cases, necrosis was observed in the largest lesion. In comparison, the largest individual lesion of cases without necrosis^PET^ had a significantly lower MATV (*p* = 0.0006) and SUV_max_ (*p* = 0.02), irrespective of *MYC* status (Supplementary Figure 2). Bulky disease was observed in 24 patients (39%). Bulky disease was significantly correlated with necrosis^PET^ (*p* = 0.005), but not with *MYC* status (*p* = 0.9) or necrosis^Hist^ (*p* = 0.8). Extranodal growth of lesions was not significantly correlated with the presence of necrosis^PET^ (*p* = 0.26).

### Survival analysis

The median follow-up was 34 months. At 5 years, OS was 67% (95% CI 54–83%), PFS was 65% (95% CI 53–81%) and DSS was 81% (95% CI 70–93%) for the entire cohort. Of the seven deaths unrelated to lymphoma, two were caused by metastatic adenocarcinoma, two were due to cardiac failure, one was due to acute on chronic renal failure and there were two cases of sudden deaths in patients in complete remission of DLBCL.

Results of the univariate Cox regression analysis (HR, 95% CI and *p* value) are shown in Table 3. The univariate analysis for OS identified *MYC*, NCCN-IPI and SUV_max_ single highest as associated factors. In univariate analysis for PFS, only NCCN-IPI was associated with outcome. In the univariate analysis for DSS *MYC*, NCCN-IPI, SUV_max_ single highest and necrosis^PET^ were associated. Both SUV_max_ and SUV_max_ single highest showed negative beta-coefficients throughout the univariate survival analysis.

**Table 3:** Univariate analysis of patient characteristics and semiquantitative ^18^F-FDG PET parameters on overall survival, progression-free survival and disease-specific survival

	Hazard ratio OS	95% CI	*p* value (Wald test)	Hazard ratio PFS	95% CI	*p* value (Wald test)	Hazard ratio DSS	95% CI	*p* value (Wald test)
*MYC*									
* MYC-*negative	Reference			Reference			Reference		
* MYC-*positive	2.9	1.1–7.4	0.025*	2.3	0.97–5.7	0.058	6.3	1.7–24	0.007**
NCCN-IPI									
0–3	Reference			Reference			Reference		
4–8	3.0	1.0–8.3	0.04*	3.6	1.3–10	0.013*	10.7	1.4–84	0.024*
necrosis^PET^									
Absent	Reference			Reference			Reference		
Present	1.7	0.6–4.5	0.3	1.8	0.7–4.6	0.2	3.9	1.2–13	0.025*
SUV_max_									
< Median	Reference			Reference			Reference		
≥ Median	0.4	0.1–1.1	0.08	0.4	0.2–1.1	0.08	0.2	0.05–1.1	0.06
SUV_max_ single highest									
< Median	Reference			Reference			Reference		
≥ Median	0.3	0.09–0.9	0.026*	0.4	0.2–1.1	0.07	0.1	0.01–0.8	0.028*
MATV									
< Median	Reference			Reference			Reference		
≥ Median	1.1	0.4–2.7	0.9	1.3	0.5–3.1	0.59	2.8	0.7–10.6	0.14
Single lesion MATV^†^									
< Median	Reference			Reference			Reference		
≥ Median	1.2	0.5–3.2	0.69	1.5	0.6–3.7	0.39	2.5	0.6–9.6	0.19
TLG									
< Median	Reference			Reference			Reference		
≥ Median	0.6	0.2–1.6	0.31	0.8	0.3–1.9	0.57	1.1	0.3–3.8	0.84

^†^Volume of the single largest/necrotic lesion; * = significance level of *p* < 0.05; ** = significance level of *p* < 0.01

**Fig. 3:**
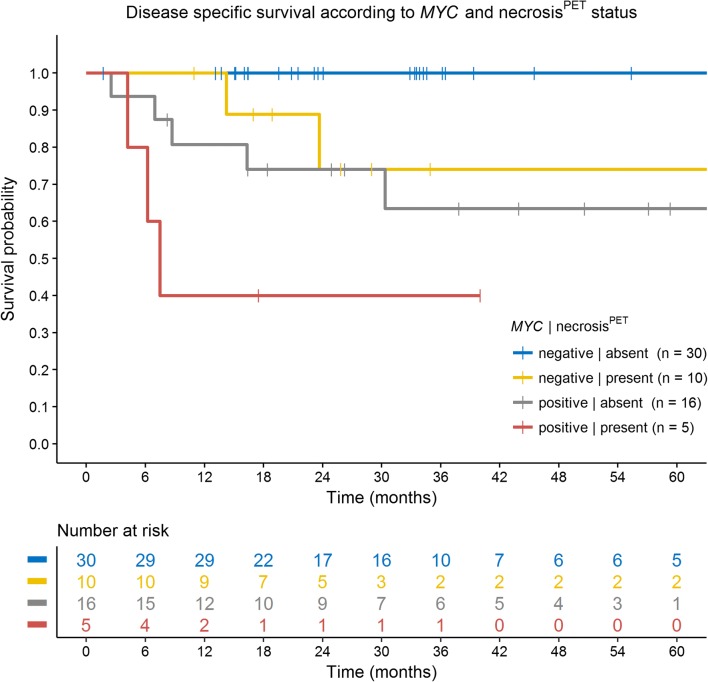
Kaplan-Meier curve showing disease-specific survival according to combined analysis with *MYC* rearrangement status and necrosis^PET^ (log-rank test, *p* = 0.00022). No events were observed in patients without *MYC* rearrangement and who had no necrosis^PET^

For multivariate analysis, the parameters *MYC*, NCCN-IPI, necrosis^PET^ and SUV_max_ single highest were used due to their prognostic impact on lymphoma-related deaths in univariate analysis (Table 4). Necrosis^PET^ did not contribute to the prognostic model for OS and PFS. However, for DSS, necrosis^PET^ had a large adverse prognostic impact and proved to be independent (HR = 13.9; 95% CI 3.0–65; *p* = 0.001). The Kaplan-Meier analysis for DSS showed no events during the 5-year follow-up period for patients who neither had *MYC* rearrangements nor had necrosis^PET^ (*n* = 30) (Fig. 3).

**Table 4:** Multivariate analysis of patient characteristics on overall survival, progression-free survival and disease-specific survival

	Hazard ratio OS	95% CI	*p* value (Wald test)	*p* value model (Wald test)
*MYC*				0.004
* MYC*-negative	Reference			
* MYC-*positive	3.1	1.1–8.7	0.029*	
NCCN-IPI				
0–3	Reference			
4–8	2.4	0.8–6.9	0.116	
necrosis^PET^				
Absent	Reference			
Present	2.6	0.9–7.7	0.079	
SUV_max_ single highest				
< Median	Reference			
≥ Median	0.3	0.1–0.9	0.027*	
	Hazard ratio PFS	95% CI	*p* value (Wald test)	*p* value model (Wald test)
*MYC*				0.005
* MYC*-negative	Reference			
* MYC-*positive	2.4	0.9–6.3	0.07	
NCCN-IPI				
0–3	Reference			
4–8	3.2	1.1–9.0	0.028*	
necrosis^PET^				
Absent	Reference			
Present	2.6	1.0–7.0	0.06	
SUV_max_ single highest				
< Median	Reference			
≥ Median	0.4	0.2–1.1	0.08	
	Hazard ratio DSS	95% CI	*p* value (Wald test)	*p* value model (Wald test)
*MYC*				0.0007
* MYC*-negative	Reference			
* MYC-*positive	14.6	2.6–82	0.002**	
NCCN-IPI				
0–3	Reference			
4–8	6.5	0.6–66	0.113	
necrosis^PET^				
Absent	Reference			
Present	13.3	2.8–63	0.001**	
SUV_max_ single highest				
< Median	Reference			
≥ Median	0.12	0.01–1.2	0.075	

* = significance level of *p* < 0.05; ** = significance level of *p* < 0.01

## Discussion

Based on the current investigation, there is no association of *MYC* rearrangements with the presence of tumour necrosis assessed by ^18^F-FDG PET or the semiquantitative ^18^F-FDG PET parameters SUV_max_, SUV_max_ single highest, MATV and TLG. We therefore rejected the hypothesis that metabolic changes induced by *MYC* rearrangements might increase the incidence of necrosis^PET^ or alter the profile of semiquantitative parameters in DLBCL. Necrosis^PET^ was significantly associated with the MATV of the single largest tumour lesion. The SUV_max_ of the single largest necrosis^PET^ lesion was significantly higher compared with the lesions without necrosis^PET^. Both of these observations support the notion of larger, more metabolically active tumours being more susceptible to necrosis, irrespective of *MYC* status.

Our analyses demonstrate that necrosis^PET^ had a significant impact on DSS, thereby substantiating previous findings about the prognostic value of this visual marker [15]. The presented data show that the presence of *MYC* rearrangement, in itself a powerful predictive factor, is not related to necrosis^PET^. This allows for integration of *MYC* status and necrosis^PET^ into a prognostic model for DLBCL. When combined with *MYC*, NCCN-IPI and SUV_max_ single highest in the multivariate analysis, necrosis^PET^ had the highest significance in predicting death due to lymphoma and a higher prognostic impact than NCCN-IPI, the currently most accurate prognostic index for DLBCL [22]. Thus, our results support the potential additive value of necrosis^PET^ as an important biomarker for risk stratification in the clinical setting [14, 15].

The lack of a relationship between *MYC* rearrangements and semiquantitative ^18^F-FDG PET metrics might have several causes. First, proliferation in DLBCL could be independent of *MYC* rearrangement. This would only partially explain the lack of relationship, since the median proliferation index (Ki-67 staining) of *MYC*^*+*^ DLBCL is universally high (> 90%) in contrast to the much broader range observed in *MYC*^*−*^ DLBCL [29]. Second, overexpression of MYC via other mechanisms such as epigenetic pathways might explain increased glucose uptake in *MYC* FISH–negative DLBCL. This is supported by studies showing high MYC protein expression in 19–40% of DLBCL cases [30–32]. Cottereau et al previously reported a lack of relation between MYC protein expression and ^18^F-FDG PET parameters in DLBCL [19]. However, FISH analysis, which is considered the gold standard examination for *MYC* rearrangements [33–35], was not performed. Third, high metabolic activity might be induced by alternative changes in metabolic drivers, such as mutations in PTEN (observed in approximately 15% of DLBCL) that lead to activation of the P13K/AKT/mTOR pathway [29, 36–38].

Intriguingly, the univariate survival analysis indicated a protective effect for cases with SUV_max_ and SUV_max_ single highest measurements above the median. Studies on the prognostic impact of these variables are conflicting [20, 39–41]. Gallicchio et al published results similar to ours, alluding to lymphomas with high metabolic activity being more responsive to chemotherapy [20]. In light of conflicting data on the prognostic value of semiquantitative ^18^F-FDG PET parameters [19–21, 42, 43], our results underline the need for larger, prospective studies with external validation cohorts [42].

This study has several limitations. First there is a referral bias with a high incidence of *MYC*^*+*^ cases (34%) in our dataset. The enrichment in our study can largely be explained by the fact that, as a reference centre, aggressive and *MYC*^*+*^ DLBCL cases (including suspected cases of Burkitt lymphoma which subsequently prove to be *MYC*^*+*^ DLBCL) are referred to our site. Second, the total number of cases with necrosis^PET^ is small, which increases the risk of a sampling error. Nevertheless, the incidence of necrosis^PET^ in our study is in line with previous studies [13–15]. Furthermore, patients were included irrespective of their comorbidities. Factors like differences in treatment regimen and non-cancer-related deaths might thus have a large impact on the statistical analysis. This is supported by the difference between DSS and OS. Despite its limitations, the prognostic potential of *MYC* status and NCCN-IPI was reproduced in this dataset, making it a representative set of DLBCL cases. Larger prospective studies are warranted to validate the prognostic value of necrosis^PET^.

## Conclusion

In this comprehensive analysis of *MYC* rearranged DLBCL, we showed that a fundamental pathological change such as *MYC* rearrangement, which by itself has a significant impact on prognosis, has no influence on the presence of necrosis^PET^ or semiquantitative ^18^F-FDG PET metrics. An explorative survival analysis suggests that the presence of necrosis determined by visual assessment of ^18^F-FDG PET scans is an independent predictor of disease-specific survival in patients with DLBCL, regardless of *MYC* status.

## Electronic supplementary material

ESM 1 (DOCX 69 kb)

## Abbreviations and acronyms

^18^F-FDG: ^18^F-fluorodeoxyglucose
B-NHL: B cell non-Hodgkin lymphoma
CT: Computed tomography
DLBCL: Diffuse large B cell lymphoma
DSS: Disease-specific survival
FISH: Fluorescence in situ hybridisation
LDH: Lactate dehydrogenase
MATV: Metabolically active tumour volume (sum of all lesions within an individual patient)
NCCN-IPI: National Comprehensive Cancer Network international prognostic index
necrosis^Hist^: Necrosis as assessed by histological scoring
necrosis^PET^: Necrosis as assessed by ^18^F-FDG PET
OS: Overall survival
PET: Positron emission tomography
PFS: Progression-free survival
SUV: Standard uptake value
SUV_max_: Highest SUV per voxel within 1 lymphoma lesion (reported here as the mean of SUV_max_ of all lesions within an individual patient)
SUV_max_ single highest: Highest SUV_max_ of all lesions within an individual patient
TLG: Total lesion glycolysis (sum of all lesions within an individual patient)
WHO: World Health Organization.
