# Supplementary material for: Tumour necrosis as assessed with 18F-FDG PET is a potential prognostic marker in diffuse large B cell lymphoma independent of MYC rearrangements
Source: Eur Radiol. 2019 Apr 26;29(11):6018–28. doi: 10.1007/s00330-019-06178-9 (PMC6795618; doi:10.1007/s00330-019-06178-9)

**Supplementary Figure 1**

Venn diagram showing the overlap between necrosis^PET^ and necrosis^Hist^ scoring indicating poor concordance.


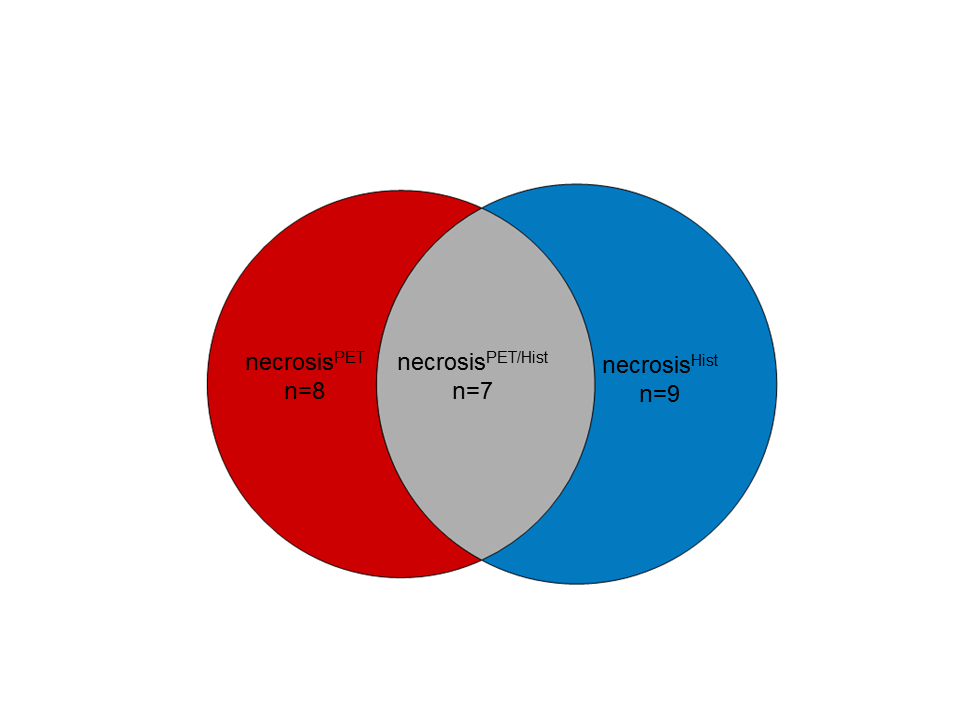


**Supplementary Figure 2**

Distribution of metabolically active tumour volume (MATV) and maximum standard uptake value (SUV_max_) of single largest tumour lesions in relation to the absence or presence of necrosis^PET^. MATV (*P* = 0.0006) and SUV_max_ (*P* = 0.02) were significantly higher in patients with necrosis^PET^. MATV and SUV_max_ showed no significant difference between *MYC* groups.


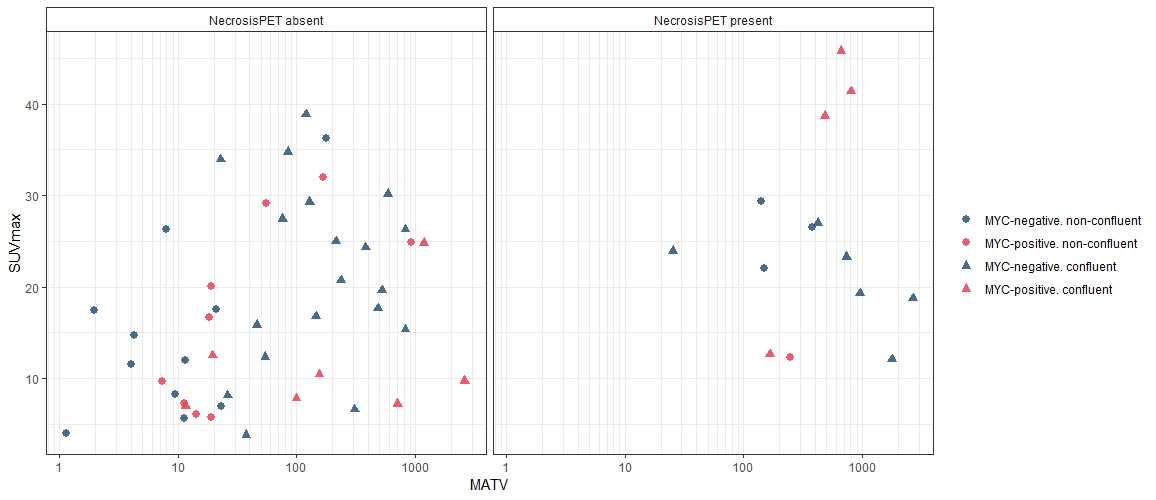

Supplement: Supplementary file 1 — (DOCX 69 kb) [file 330_2019_6178_MOESM1_ESM.docx]
